# Is the heart rate variability monitoring using the analgesia nociception index a predictor of illness severity and mortality in critically ill patients with COVID-19? A pilot study

**DOI:** 10.1371/journal.pone.0249128

**Published:** 2021-03-24

**Authors:** Cristian Aragón-Benedí, Pablo Oliver-Forniés, Felice Galluccio, Ece Yamak Altinpulluk, Tolga Ergonenc, Abdallah El Sayed Allam, Carlos Salazar, Mario Fajardo-Pérez

**Affiliations:** 1 Department of Anesthesia, Resuscitation and Pain Therapy, Mostoles General University Hospital, Mostoles, Madrid, Spain; 2 Morphological Madrid Research Center (MoMaRC), Ultradissection Spain EchoTraining School, Madrid, Spain; 3 Department of Anesthesia, Resuscitation and Pain Therapy, Lozano Blesa University Clinic Hospital, Zaragoza, Aragón, Spain; 4 Department of Clinical and Experimental Medicine, University Hospital AOU Careggi, Florence, Italy; 5 Outcomes Research Department, Anesthesiology Institute, Cleveland Clinic, Cleveland, OH, United States of America; 6 Department of Anesthesiology and Reanimation, Cerrahpasa Medical Faculty, Istanbul University-Cerrahpasa, Istanbul, Turkey; 7 Anesthesiology Clinical Research Office, Ataturk University, Erzurum, Turkey; 8 Department of Anesthesiology, Akyazi Pain and Palliative Care Center, Sakarya, Turkey; 9 Sakarya Education and Research Hospital, Sakarya, Turkey; 10 Department of Physical Medicine, Rheumatology and Rehabilitation, Faculty of Medicine and University Hospital, Tanta University, Tanta, Egypt; 11 Department of Anesthesia, Hospital Universitario 12 de Octubre, Madrid, Spain; Heidelberg University Hospital, GERMANY

## Abstract

**Introduction:**

The analysis of heart rate variability (HRV) has proven to be an important tool for the management of autonomous nerve system in both surgical and critically ill patients. We conducted this study to show the different spectral frequency and time domain parameters of HRV as a prospective predictor for critically ill patients, and in particular for COVID-19 patients who are on mechanical ventilation. The hypothesis is that most severely ill COVID-19 patients have a depletion of the sympathetic nervous system and a predominance of parasympathetic activity reflecting the remaining compensatory anti-inflammatory response.

**Materials and methods:**

A single-center, prospective, observational pilot study which included COVID-19 patients admitted to the Surgical Intensive Care Unit was conducted. The normalized high-frequency component (HFnu), i.e. ANIm, and the standard deviation of RR intervals (SDNN), i.e. Energy, were recorded using the analgesia nociception index monitor (ANI). To estimate the severity and mortality we used the SOFA score and the date of discharge or date of death.

**Results:**

A total of fourteen patients were finally included in the study. ANIm were higher in the non-survivor group (p = 0.003) and were correlated with higher IL-6 levels (p = 0.020). Energy was inversely correlated with SOFA (p = 0.039) and fewer survival days (p = 0.046). A limit value at 80 of ANIm, predicted mortalities with a sensitivity of 100% and specificity of 85.7%. In the case of Energy, a limit value of 0.41 ms predicted mortality with all predictive values of 71.4%.

**Conclusion:**

A low autonomic nervous system activity, i.e. low SDNN or Energy, and a predominance of the parasympathetic system, i.e. low HFnu or ANIm, due to the sympathetic depletion in COVID-19 patients are associated with a worse prognosis, higher mortality, and higher IL-6 levels.

## Introduction

Since the pandemic started in early 2020, critically ill patients suffering from COVID-19 on mechanical ventilation have become one of the most challenging problems in intensive care units around the world [1].

As it is suggested [2], there are three stages in the COVID-19 disease: the first stage of viral replication, the second stage of lung involvement, with the development of severe pneumonia and ARDS [3], and the third stage with a predominance of a hyper-immune response, with severe multi-organ dysfunction.

What happens in the third stage is a severe inflammatory response syndrome (SIRS), also known as cytokine release syndrome (CRS), with extreme macrophage activation and a significant increase in inflammatory cytokines, such as Interleukin (IL-6), ferritin, C-reactive protein (CRP) or D-dimer [4].

This strong hyper-immune reaction produces a large adrenergic release, which is mainly modulated by the sympathetic nervous system [5, 6]. This macrophage activation syndrome is in turn balanced by a compensatory anti-inflammatory response (CARS), which is mostly modulated by the anti-inflammatory cholinergic pathway and the parasympathetic nervous system [7, 8].

Thus, the autonomic nervous system (ANS) is responsible for the regulation of this inflammatory reflex, and its balance is essential to the maintenance of the body’s homeostasis [9, 10]. A physiological metric for the measurement of the ANS is the analysis of heart rate variability (HRV) and, more specifically, the analgesia nociception index (ANI) monitor has proven to be a crucial tool for the measurement of the ANS and nociception in both surgical and critically ill patients [11–15].

It is hypothesized that a high level of normalized high-frequency (HFnu) component of HRV and a low standard deviation of normal-to-normal RR intervals (SDNN) could have a predictive value in terms of severity and mortality in critically ill patients suffering from COVID-19; furthermore, it is also hypothesized that these values might be related to the number of proinflammatory cytokines, such as IL-6, CRP, and procalcitonin.

The main objective is to demonstrate that the most severely ill COVID-19 patients will show greater dysregulation of the ANS, with a significant depletion of the sympathetic nervous system and a slight predominance of parasympathetic activity, reflecting the remaining compensatory anti-inflammatory response.

## Material and methods

### Study design and setting

A single-center, prospective, observational, pilot study was designed, which included COVID-19 patients admitted to the Surgical Intensive Care Unit of the Mostoles General University Hospital in Madrid between April and May 2020. The reporting of this study conforms to the STROBE statement.

### Ethics

The study was performed in line with the principles of the Declaration of Helsinki and it was approved by the Ethical and Research Committee of Mostoles General University Hospital, with registration code No. 2020/035. In the light of the COVID-19 pandemic to avoid the risk of viral transmission and following current ethical and legal recommendations, verbal informed consent was obtained from all subjects by the legal designees and recording the date and time in the case record form and the patient’s clinical history, which was justified and approved by the Ethical and Research Committee.

### Inclusion/Exclusion criteria

The inclusion criteria were defined as follows: patients over 18 years of age, on mechanical ventilation, through orotracheal intubation or tracheostomy, diagnosed with COVID-19 by a positive polymerase chain reaction (PCR) test for SARS-COV-2. Exclusion criteria included: patients with the use of pacemakers, a history of cardiac arrhythmia, or without normal sinus rhythm.

### Heart rate variability

HRV refers to the variation between one heartbeat and the next, i.e. R-R interval on an ECG, a process that is influenced by different components of the ANS, including breathing and other physiological factors [11, 12, 16]. Inhalation temporarily inhibits the influence of the parasympathetic nervous system and increases heart rate, while exhalation stimulates the parasympathetic nervous system and decreases heart rate. These rhythmic oscillations, which are caused by breathing, are called respiratory sinus arrhythmia (RSA) [17].

Spectral density analysis of the different frequency and time domain indices of HRV is a non-invasive method that evaluates the activity of the ANS [11, 12]. The high-frequency (HF) component, between 0.15 Hz and 0.4 Hz, is mediated by the parasympathetic nervous system and breathing; the low-frequency (LF) component, between 0.15 to 0.04 Hz, is mainly influenced by the sympathetic nervous system and baroreflex mechanisms; and, lastly, the very-low-frequency (VLF) component, between 0.04 to 0.003 Hz, is influenced by thermoregulation and different hormonal factors [9]. The standard deviation of all normal R–R intervals (SDNN), SDNN expresses the overall HRV and reflects the overall activity of the ANS, calculated in milliseconds (ms). The correlation between SDNN and total power (TP) is well described in the HRV literature, which is a short-term estimate of the total power spectral density in the range of frequencies between 0 and 0.4 Hz (HF+LF+VLF) and represents also the ANS activity, calculated in milliseconds squared (ms^2^). Normalized unit spectral indices of HF (HFnu) are usually defined as the ratio between the absolute value of the HF and the SDNN or TP (HFnu = HF / (HF + LF + VLF). It is calculated in percentile units and reflects the modulation of the parasympathetic branch of the ANS [17–19].

### Analgesia nociception index

The parameters of HRV were recorded using the analgesia nociception index monitor (ANI monitor, MDoloris Medical Systems, Lille, France). ANI is an index for calculating the HFnu. It is done by a graphical method calculating the area under the curve of the sinusoid obtain from the HRV produced by the RSA, as it has been described by Logier R et al. and Jeanne M et al. [18, 19].

Thus, the ANI monitor provides a number from 0 to 100, which represents the HFnu, i.e. the percentage estimate of the balance between the parasympathetic nervous system and the added activity of the different spectral components [17–19]. The mean ANI of the last 240 seconds is represented by the ANIm value and the instant ANI of the last 120 seconds is represented by the ANIi value. Furthermore, the ANI monitor shows the value of the SDNN by the term “Energy”. These are the mathematical formulas for Energy, where RRmoy is the average of R-R and N is the number of R-R:

### Measurements and data handling

The ANIm, ANIi and mean Energy values were collected for 240 seconds, from a single measurement in the morning before daily washing. During the study period, changes in drugs potentially affecting HRV and invasive procedures were avoided. For this, the specific ANI monitor electrodes for ECG were placed on the patient’s chest or back, depending on whether they were in a supine or prone position.

As demographic data, the age, sex, and weight of the patients were recorded. Drugs used for sedoanalgesia, neuromuscular blocking drugs, and the need for vasoactive drugs (norepinephrine and/or dobutamine) were also recorded.

Sedoanalgesia was maintained using continuous infusions of different drugs, according to usual department protocol. This protocol includes midazolam (0.03–0.2 mg.kg.hour^-1^), morphine (0.5–5 mg.hour^-1^), propofol (0.5–4 mg.kg.hour^-1^), remifentanil (0.05–0.2 mcg.kg.min^-1^), and/or dexmedetomidine (0.4–1.4 mcg.kg.min^-1^). In cases where neuromuscular blocking was needed, it was used a continuous perfusion of cisatracurium (0.06–0.3 mg.kg.hour^-1^) or rocuronium (0.3–0.6 mg.kg.hour^-1^).

To assess the degree of sedation, it was used the Richmond Agitation-Sedation scale (RASS) [12], and to estimate the severity, it was used the SOFA score (Sequential Organ Failure Assessment), validated for critically ill patients [20], as well as data for IL-6, CRP, and procalcitonin. A blood test was performed in the morning of the same day of the HRV measure and the values were obtained from the central hospital laboratory.

Also, data on ventilatory parameters (ventilatory mode, tidal volume, respiratory rate, and positive pressure at the end of expiration [PEEP]) were collected. Mechanical ventilation was personalized for each patient according to the severity of illness and gasometric analytical parameters, as per the usual department protocols.

Subsequently, within 30 days after all these data collection, the patient’s electronic medical record was checked and the date of hospital admission, date of admission to ICU, and date of discharge to hospital facility, survival days, or date of death were recorded. According to the 30 days mortality after data collection, the patients were categorized into non-survivor group and survivor group.

### Sample size

It was determined 14 patients at the beginning of the pilot study to test feasibility protocols adherence and data collection. In terms of post-hoc power calculations, a sample size of 7 subjects per group (survived, not survived) yields 94% power to declare a significant difference in the distribution of ANIm and Energy scores assuming the medians and quartiles estimated in the study.

### Statistical analysis

To analyze the data, non-parametric tests were used. In the descriptive analysis of the data, the median and quartiles (first and third quartiles) were used. For the study of homogeneity of the sample and comparison of medians, the U-Mann-Whitney and Wilcoxon tests were performed. Kendall’s tau-b correlation test was used to detect the bivariate relationship between variables. To find a threshold value to attempt to predict the risk of mortality and for the calculation of diagnostic accuracy, the corresponding receiver operating characteristic (ROC) curves were analyzed for both the ANIm value and the Energy value. There was no adjustment for multiple comparisons given the pilot nature of the study.

P-values <0.05 were considered to be statistically significant. Apple Numbers version 10.3.9 was used to collect data, and the different analyses were carried out using commands from the basic “stats” package of Software “R”, version 3.1.2. The data that support the findings of this study are available Open Access.

## Results

**Fig 1:**
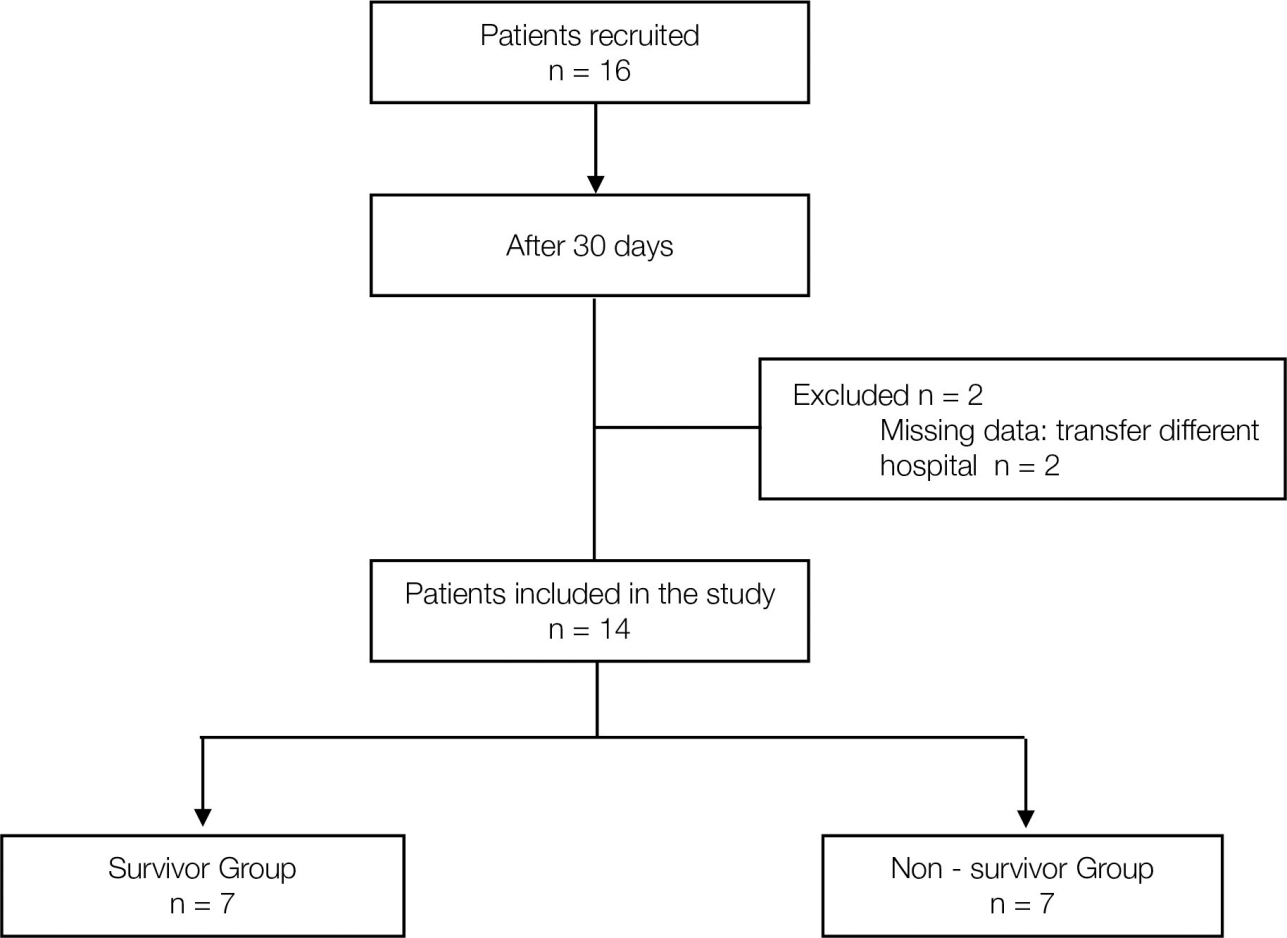
STROBE patient flow diagram.

During the data collection period, 16 patients were recruited, 2 of whom were excluded as detailed in Fig 1. STROBE patient flow diagram. A total of 14 patients were finally included in the study, with 7 patients belonging to the survivor group and 7 others belonging to the non-survivor group.

The differences between the groups in patient demographic, sedoanalgesia, and ventilatory parameters data are shown in Table 1. The only differences in the groups were in terms of the use of neuromuscular blockers (p = 0.029), the RASS scale (p = 0.021) and PEEP value (p = 0.032). Also, the SOFA scores between the two groups were statistically different (p = 0.031).

**Table 1:** Homogeneity and comparison of demographic and characteristics data between groups.

	Survivor Group n = 7	Non—Survivor Group n = 7	p-value
Sex (Male)	4 (57%)	7 (100%)	0.192
Sex (Female)	3 (43%)	0 (0%)	
Age (years)	64 (60; 73)	71 (57; 72)	0.797
Weight (kg)	74 (68; 78)	85 (75; 99)	0.085
SOFA	3 (2; 6)	8 (3; 9)	0.032
RASS	-4 (-4; -2)	-5 (-5; -4)	0.021
CRP (mg.dl^-1^)	51 (2.2; 197)	112 (26; 309)	0.482
IL-6 (pg.ml^-1^)	145 (111; 567)	963 (702; 1350)	0.055
Procalcitonin (ng.ml^-1^)	0.6 (0.37; 0.65)	0.33 (0.12; 0.68)	0.370
Midazolam	3 (43%)	5 (71%)	0.592
Propofol	4 (57%)	1 (14%)	0.266
Dexmedetomidine	2 (29%)	2 (29%)	1.000
Fentanil	2 (29%)	1 (14%)	1.000
Remifentanil	0 (0%)	2 (29%)	0.462
Morphine	3 (43%)	4 (57%)	1.000
Lidocaine	3 (43%)	1 (14%)	0.559
Neuromusc. blockade	1 (14%)	6 (86%)	0.029
Noradrenaline	0 (0%)	3 (43%)	0.192
Dobutamine	1 (14%)	0 (0%)	1.000
VCV mode	3 (43%)	6 (86%)	0.266
PCV mode	4 (57%)	1 (14%)	
FiO2	0.7 (0.7; 0.8)	1 (0.8; 1)	0.009
Tidal Volume (ml)	470 (450; 480)	480 (450; 520)	0.478
Resp. rate (rpm)	18 (18; 20)	20 (18; 20)	0.375
PEEP (cmH_2_O)	8 (6; 10)	10 (9; 12)	0.032
PaO2 (mmHg)	143 (95.6; 301)	124 (81.2; 137)	0.225
ANIm	64 (53; 74)	93 (89; 99)	0.003
ANIi	64 (57; 76)	94 (87; 99)	0.006
Energy (ms)	0.57 (0.3; 0.63)	0.18 (0.13; 0.71)	0.225

Basic descriptive and tests for the demographic and characteristics variables for each group. Absolute (N) and relative (%) frequencies for the qualitative variables, and median and quartiles (1st; 3rd) for the quantitative variables. P-values were calculated using Mann—Whitney U test. SOFA, Sequential Organ Failure Assessment; RASS, Richmond Agitation-Sedation Scale; CRP, C-reactive protein; IL-6, Interleukin-6; VCV, Volume-controlled ventilation; FiO2, Fraction of inspired oxygen; PEEP, positive end-expiratory pressure; PaO2, partial pressure of oxygen in arterial blood; ANIm, mean analgesia nociception index; ANIi, instantaneous analgesia nociception index.

### Analgesia nociception index and Energy

**Fig 2:**
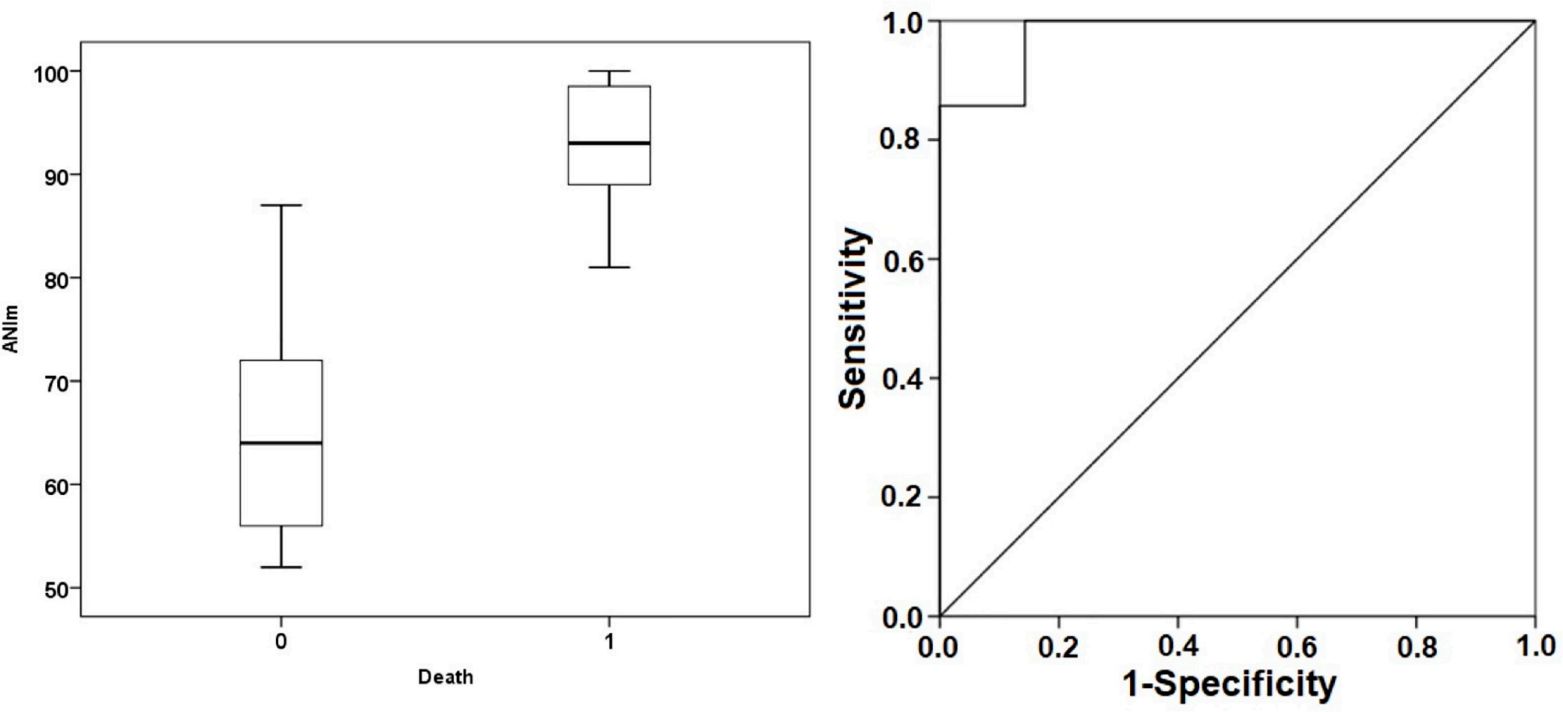
Box plot (left) and ROC curves for ANIm (right). Box plot represents the median values of ANIm in both groups. ROC curve demonstrates the ability of ANI to discriminate the mortality with an AUC = 0.980 at an ANIm threshold of 80 (sensitivity 100%, specificity 85.7%, positive predictive value 87.5%, negative predictive value 100%). ANIm, median analgesia nociception index; 0 death, survivor group; 1 death, non-survivor group.

The ANIm figures were considerably higher in the deceased group, with statistically significant differences. A Mann-Whitney test indicated that the ANIm value was higher for the non-survivor group 93% (89; 99) than for the survivor group 64% (53; 74) with p = 0.003 (Fig 2).

**Fig 3:**
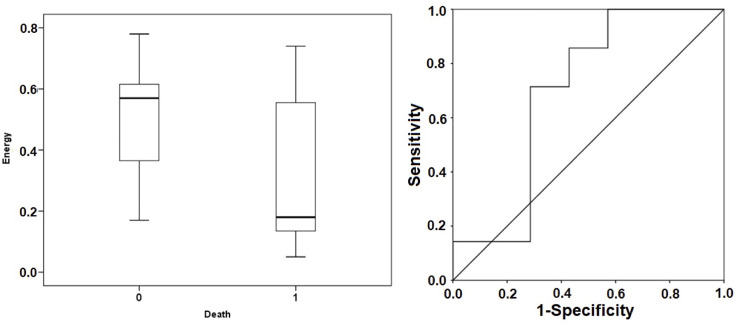
Box plot (left) and ROC curves for Energy (right). Box plot represents the median values of Energy in both groups. ROC curve demonstrates the ability of Energy to discriminate the mortality with an AUC = 0.694 at a threshold of 0.41 ms (sensitivity 71.4%, specificity 71.4%, positive predictive value 71.4%, negative predictive value 71.4%). 0 death, survivor group; 1 death, non-survivor group.

However, in terms of the Energy figures, although lower in the non-survivor group (Fig 3), there were no statistically significant differences. A Mann-Whitney test indicated that the Energy value was not different for the non-survivor group 0.18 ms (0.13; 0.71) from the survivor group 0.57 ms (0.3; 0.63) with p = 0.225.

Looking closer at the correlation between ANIm and Energy with respect to the SOFA score, it was discovered that the ANIm value was not statistically correlated (p = 0.179, Kendall’s tau-b test). However, the Energy itself was inversely correlated with the SOFA score (p = 0.039, Kendall’s tau-b test). In other words, patients with lower Energy presented with greater severity of illness and a worse prognosis.

On the other hand, when analyzing inflammatory cytokines (IL-6, PCR, procalcitonin), it was discovered that the Energy levels were not statistically correlated with any of them. However, higher ANIm levels were statistically correlated with higher IL-6 levels (p = 0.020, Kendall’s tau-b test). There was no such relationship with the rest of the cytokines, such as PCR (p = 0.546, Kendall’s tau-b test) or procalcitonin (p = 0.912, Kendall’s tau-b test).

For the ANIm value, we found that a limit value of 80 predicted mortalities with a sensitivity of 100%, a specificity of 85.7%, a positive predictive value of 87.5%, and a negative predictive value of 100% (Fig 2). In the case of Energy, a limit value of 0.41 ms predicted mortality with a sensitivity of 71.4%, a specificity of 71.4%, a positive predictive value of 71.4%, and a negative predictive value of 71.4% (Fig 3).

If it is looked specifically at the non-survivor group, it was found not only that the Energy and the SOFA scores correlated (p = 0.009, Kendall’s tau-b test), but that patients with lower Energy values had fewer survival days (p = 0.046, Kendall’s tau-b test).

### Sub-analysis in RASS - 4 / - 5 patients

Although the distribution of drugs between the groups was homogeneous (Table 1), to minimize the bias that may occur between ANI monitor values, drug dosage, and the RASS, a sub-analysis was carried out only for patients with RASS -4/ -5. Three patients in the survivor group were removed from the sub-analysis, Fig 1. STROBE patient flow diagram. In this way, all existing differences in homogeneity between the groups were eliminated (Table 2).

**Table 2:** Sub-analysis for Richmond agitation-sedation scale—4 / - 5 patients: Homogeneity and comparison of demographic data and characteristics between groups.

	Survivor Group n = 4	Non—Survivor Group n = 7	p-value
Sex (Male)	2 (50%)	7 (100%)	0.109
Sex (Female)	2 (50%)	0 (0%)	
Age (years)	62 (60; 71)	71 (57; 72)	0.788
Weight (kg)	78 (73; 91)	85 (75; 99)	0.527
SOFA	2.5 (2; 6)	8 (3; 9)	0.055
RASS	-4 (-4.75; -4)	-5 (-5; -4)	0.156
CRP (mg.dl^-1^)	6.1 (2.2; 188.5)	112 (26; 309)	0.256
IL-6 (pg.ml^-1^)	153.3 (0.24; 1.65)	963 (702; 1350)	0.439
Procalcitonin (ng.ml^-1^)	0.6 (0.24; 1.65)	0.33 (0.12; 0.68)	0.506
Midazolm	3 (75%)	5 (71%)	1.000
Propofol	1 (25%)	1 (14%)	1.000
Dexmedetomidine	1 (25%)	2 (29%)	1.000
Fentanil	1 (25%)	1 (14%)	1.000
Remifentanil	0 (0%)	2 (29%)	0.491
Morphine	2 (50%)	4 (57%)	1.000
Lidocaine	2 (50%)	1 (14%)	0.491
Neuromusc. blockade	1 (25%)	6 (86%)	0.088
Noradrenaline	0 (0%)	3 (43%)	0.236
Dobutamine	0 (0%)	0 (0%)	1.000
VCV mode	3 (75%)	6 (86%)	1.000
PCV mode	1 (25%)	1 (14%)	
FiO2	0.75 (0.7; 0.8)	1 (0.8; 1)	0.017
Tidal Volume (ml)	480 (420; 510)	480 (450; 520)	0.848
Resp. rate (rpm)	18 (16.5; 18)	20 (18; 20)	0.067
PEEP (cmH_2_O)	9 (7.25; 10)	10 (9; 12)	0.145
PaO2 (mmHg)	265.3 (129.7; 309.9)	124 (81.2; 137)	0.059
ANIm	61.5 (52.3; 73)	93 (89; 99)	0.008
ANIi	63 (58.3; 79.8)	94 (87; 99)	0.014
Energy (ms)	0.62 (0.38; 0.74)	0.18 (0.13; 0.71)	0.186

Basic descriptives and tests for the demographic and characteristics variables for each group. Absolute (N) and relative (%) frequencies for the qualitative variables, and median and quartiles (1st; 3rd) for the quantitative variables. P-values were calculated using Mann—Whitney U test. SOFA, Sequential Organ Failure Assessment; RASS, Richmond Agitation-Sedation Scale; CRP, C-reactive protein; IL-6, Interleukin-6; VCV, Volume-controlled ventilation; FiO2, Fraction of inspired oxygen; PEEP, positive end-expiratory pressure; PaO2, partial pressure of oxygen in arterial blood; ANIm, mean analgesia nociception index; ANIi, instantaneous analgesia nociception index.

**Fig 4:**
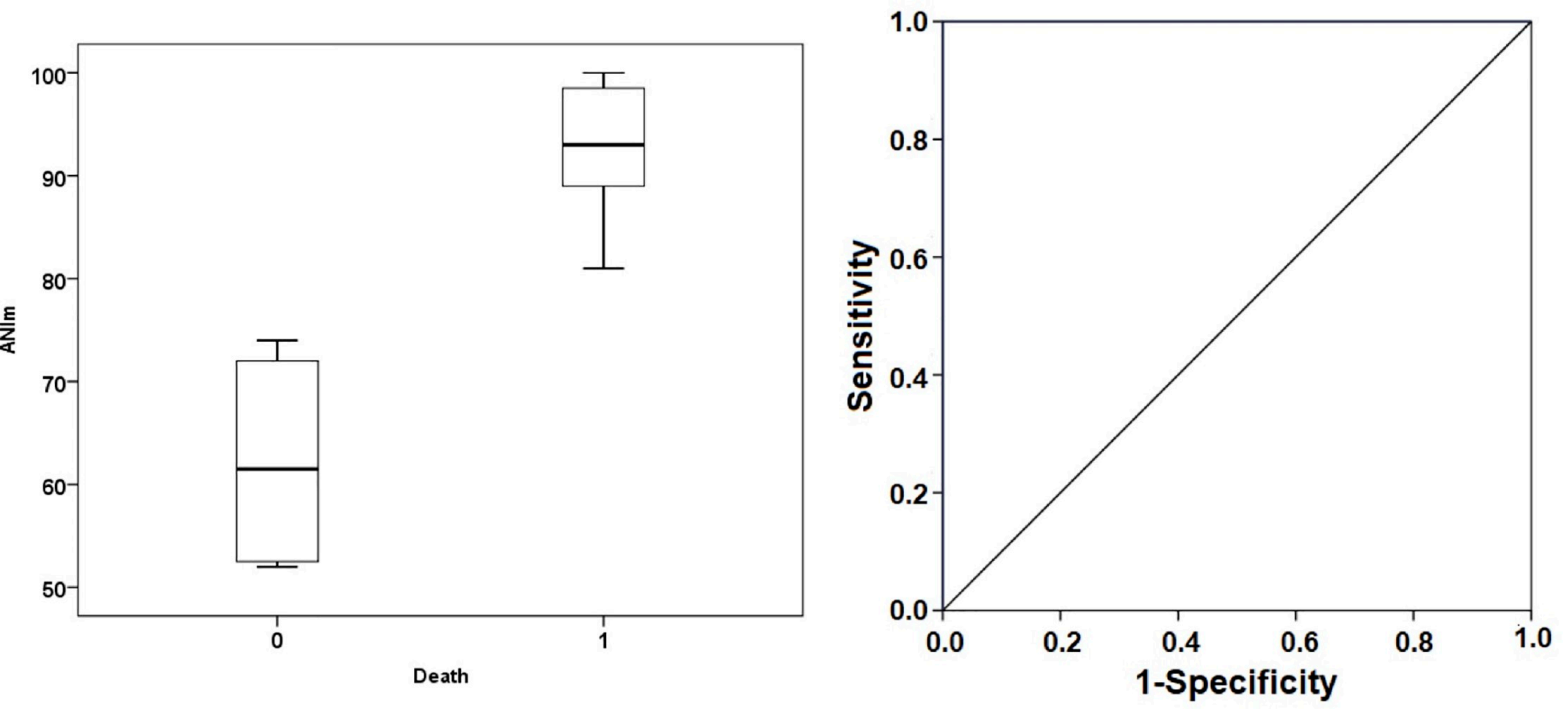
Sub-analysis: Box plot (left) and ROC curves for ANIm (right) in the RASS -4 / -5 patients. Box plot represents the median values of ANIm in both groups. ROC curve demonstrates the ability of ANI to discriminate the mortality with an AUC = 1 at an ANIm threshold of 80 (sensitivity 100%, specificity 100%, positive predictive value 100%, negative predictive value 100%). ANIm, median analgesia nociception index; 0 death, survivor group; 1 death, non-survivor group.

**Fig 5:**
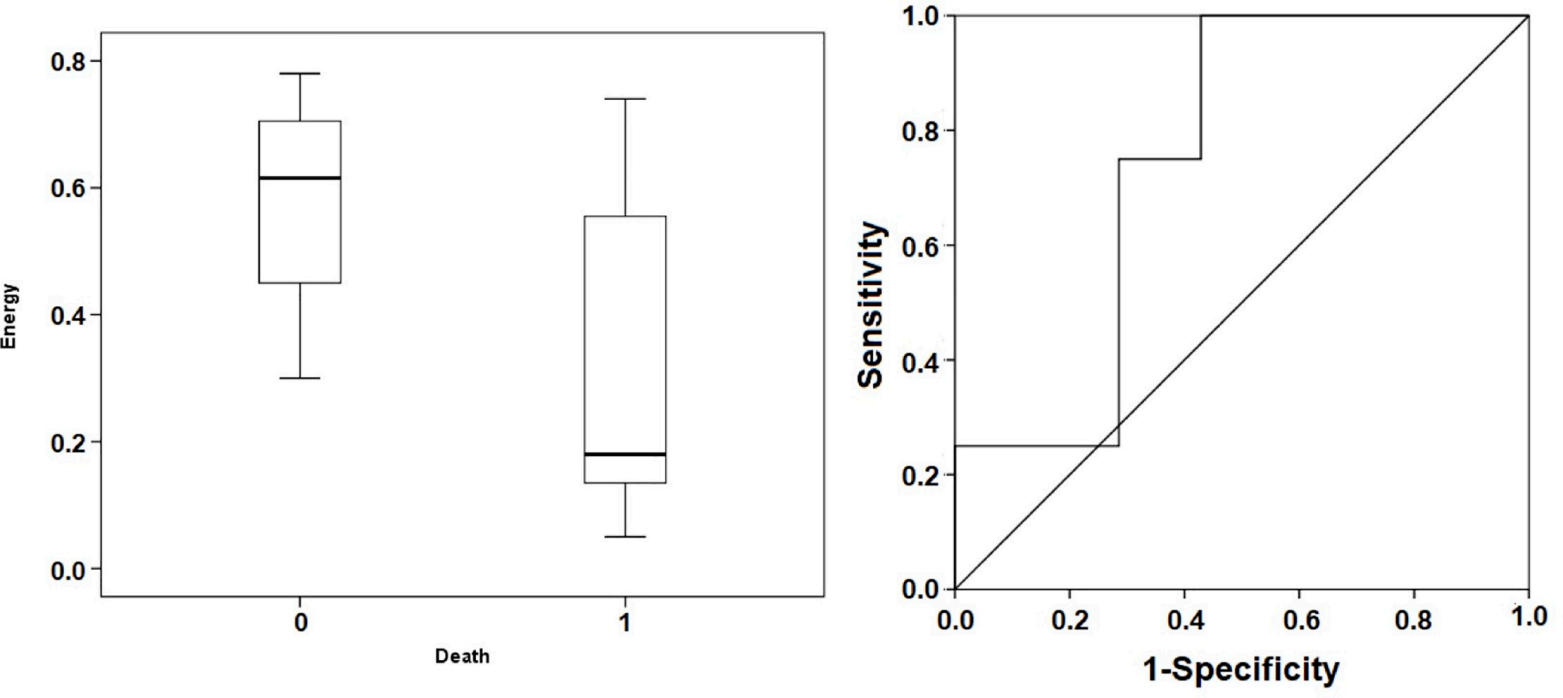
Sub-analysis: Box plot (left) and ROC curves for Energy (right) in the RASS -4 / -5 patients. Box plot represents the median values of Energy in both groups. ROC curve demonstrates the ability of Energy to discriminate the mortality with an AUC = 0.750 at a threshold of 0.41 (sensitivity 71.4%, specificity 75%, positive predictive value 83.3%, negative predictive value 60%). 0 death, survivor group; 1 death, non-survivor group.

In the sub-analysis in patients with RASS -4/-5, it was shown that the difference between groups in terms of the ANIm value and Energy was much greater and that the capacity to predict prognosis and death using these two values was higher. In the case of ANIm, all its predictive values, for a limit value of 80, were 100% (Fig 4). In terms of Energy in this group of patients, for a limit value of 0.41 ms, sensitivity was 71.4%, specificity was 75%, the positive predictive value was 83.3%, and the negative predictive value was 60% (Fig 5).

## Discussion

This prospective, observational, pilot study was intended to clarify certain questions arising in the last year about the dysregulation of the ANS in COVID-19 patients. According to the available literature, this is the first study to analyze the time and frequency domain parameters of HRV as a prospective predictor of illness severity and mortality in critically ill patients suffering from SARS-COV-2 who are on mechanical ventilation.

### Heart rate variability as a prognosis tool

Our study is consistent with the other research findings that have also analyzed HRV in critically ill patients [16, 20–23]. As seen in several studies, such as Ahmad S et al. [22] or Pontet J et al. [24], carried out especially in septic patients conclude that those with lower HRV, a reduction of the sympathetic component (LF and LFnu) and a predominance of the parasympathetic component (HF and HFnu) presented increased severity according to the APACHE II score and predicted which patients had the highest risk of developing multiple organ dysfunction syndromes (MODS) [22, 23, 25, 26].

Another study by Chen-WL et al. [27] showed that monitoring HRV at the time of admission to the emergency room for patients resuscitated after the myocardial infarction could predict 24-hour mortality. Those with the worst prognosis presented depletion of global HRV (TP or SDNN), decreased sympathovagal balance, i.e. low LFnu, and renin-angiotensin-aldosterone modulation (VLF), as compared to healthy subjects.

Also, Huang CT et al. [28] concluded that spectral analysis of HRV in 101 patients admitted to the intensive care unit undergoing mechanical ventilation could predict the success or failure of removal of said support and that patients extubated with a lower TP had a higher risk of reintubation after 72 hours. Moreover, Chen IC et al. [29] demonstrated that TP and HF power were independent predictors of mortality in patients with adult respiratory distress syndrome (ARDS) on admission to the SICU.

Similarly to these studies, according to our results, we founded that COVID-19 patients in a critical state who presented with low autonomic nervous system activity, i.e., a lower SDNN, have a worse prognosis according to the predictive SOFA score. Besides, a depletion of sympathetic activity and proportionally greater vagal activity, i.e. a high HFnu, was associated with higher mortality.

### Heart rate variability and inflammation

Concerning the HRV and inflammation, a recent meta-analysis of 51 studies [30] with a total of 2238 patients concluded that spectral analysis serves to monitor the autonomic activity that controls inflammatory processes in humans. These researchers have shown a strong association among inflammatory parameters, mainly IL-6 and CRP, and a higher high-frequency band (HF), and a low SDNN.

More specifically, Hasty F et al. [31] have recently shown that dramatic drops in HRV (SDNN) have correlated with subsequent spikes in CRP in COVID-19 patients. However, we have not been able to detect any correlation between SDNN, i.e. Energy, and IL-6, procalcitonin, or CRP, but we found that higher values of HFnu were correlated with higher IL-6 values.

### Severe inflammatory response syndrome in COVID-19

This decreased activity of the ANS, along with the increase in the parasympathetic component, seen in patients with COVID-19 and critically ill patients in general, would represent what happens in the late phase when there is significant autonomic dysregulation, with large-scale sympathetic adrenergic depletion, and a slight predominance of parasympathetic activity as a reflection of the compensatory response [32].

When SIRS subsides and CARS is active for some time, without returning to a state of homeostasis, a state of immunodeficiency or anergy is frequently produced, which triggers an increase in viral replication and bacterial superinfection and can ultimately lead to a fatal outcome for the patient [5, 6, 33, 34].

Panigrahy D et al. [35] showed in a recent study that most of the latest clinical trials on COVID-19 patients have focused primarily on "anti-viral" and "anti-inflammatory" therapeutic strategies. However, they suggested that perhaps a new therapeutic approach for more severely ill patients could be the stimulation of this innate inflammatory response.

### Implications and cholinergic anti-inflammatory pathway

The cholinergic anti-inflammatory pathway is a mechanism for neural inhibition of inflammation and interfaces the brain with the immune system [7, 9, 10, 36]. In this regard, there is a nucleus in the brain stem that directs the inflammatory reflex when any injury, infection, or nociceptive stimulus occurs, activating the autonomic nociceptive circuit described by Brown EN et al. [37, 38]. This is the nucleus of the solitary tract (NTS), which, through the vagus nerve (VN) and the activation of the different nuclei of the central nervous system, modulates both the sympathetic and parasympathetic nervous systems. The NTS activates the cholinergic anti-inflammatory pathway through the VN. The VN is a powerful anti-inflammatory element, as it releases acetylcholine, which inhibits macrophage release of cytokines by binding to its specific membrane receptor, the nicotinic alpha 7 receptor [36].

In turn, the NTS produces activation of the entire sympathetic chain through the rostral ventromedial medulla (RVM), activates the locus coeruleus (LC) nucleus that regulates the “fight or flight” response through noradrenergic release, and activates the hypothalamic-pituitary-adrenal system by releasing adrenocorticotropic hormone (ACTH) following the activation of the paraventricular (PV) nucleus of the hypothalamus [7, 9, 10, 36].

Therefore, activation of the NTS and the cholinergic anti-inflammatory pathway, both pharmacologically, by activating the alpha 7 nicotinic receptors, and electrically, through non-invasive brain neuromodulation and vagus nerve stimulation (VNS), as also suggested by Baptista et al. [39], appear to be promising therapeutic strategies to balance the ANS and produce a balanced autonomic response [40–42]. Besides, Leitzke et al. [43] have recently reported that these therapeutic approaches for sympathovagal balance in severe courses of COVID-19 can be achieved diagnostically by measuring HRV.

Several researchers have already tested VNS in patients with immune system disorders and sepsis with promising results [44, 45], and some clinical trials in COVID-19 patients have started over the last few months, such as Tornero et al. SAVIOR protocol [46, 47].

### Limitations

This is a pilot study carried out with certain real-life limitations during the pandemic. Despite using a convenience sample, one of our main limitations is the small sample of patients used.

Secondly, and in this regard, it has not been possible to perform a multivariate adjustment for the known disturbance variables. There are few patients to make an adjustment meaningful, and such baseline adjustments could not be formally made, due to the sample size.

Besides, our results were based on clinical management under real-life conditions in a single-center. We have not analyzed other factors, such as the dose of sedoanalgesia drugs, the degree of neuromuscular blockade, the use of monoclonal antibodies, such as tocilizumab, and glucocorticoids, and others that are known to contribute to modulating the immune system, and indirectly to the autonomic nervous system.

For all of these reasons, further research is required to provide more evidence and to overcome the methodological issues of this study.

In a future study, we will attempt to monitor neuromuscular blocking data using acceleromyograph, record the degree of sedoanalgesia, using electroencephalogram monitors which include spectrogram analysis, and measure the degree of pulmonary involvement measured by chest CT, lung ultrasonography, or X-ray.

## Conclusion

It may be concluded that the different components of the spectral analysis of HRV, allow us to infer the state of the autonomic nervous system and the immune system of critically ill patients. Based on the results of our study, low autonomic nervous system activity, i.e. low Energy or SDNN, and a predominance of the parasympathetic system due to sympathetic depletion, i.e. high ANI value or HFnu, are associated with a worse prognosis and higher mortality. In our critically ill patients with COVID-19 sample, a high ANIm value above 80 and a low Energy value below 0.41 ms, during admission in the ICU, especially in more sedated patients with RASS -4/-5, predicted mortality with very high sensitivity and specificity.

This autonomic dysregulation likely represents the cause and effect of the different stages of SARS-COV-2 disease, the severe inflammatory system response syndrome (SIRS), and its compensatory anti-inflammatory response (CARS). Therefore, for future studies, it is proposed that the use of non-invasive neuromodulation techniques of the autonomic nervous system may encourage a balance between the sympathetic/parasympathetic components and might be used as a therapeutic strategy in critically ill patients with COVID-19.

## Supporting information

S1 Data (XLSX)
