# Correction: Is the heart rate variability monitoring using the analgesia nociception index a predictor of illness severity and mortality in critically ill patients with COVID-19? A pilot study

**DOI:** 10.1371/journal.pone.0252259

**Published:** 2021-05-20

**Authors:** Cristian Aragón-Benedí, Pablo Oliver-Forniés, Felice Galluccio, Ece Yamak Altinpulluk, Tolga Ergonenc, Abdallah El Sayed Allam, Carlos Salazar, Mario Fajardo-Pérez

There is an error in the Conclusion subsection of the Abstract. The correct sentence is: A low autonomic nervous system activity, i.e. low SDNN or Energy, and a predominance of the parasympathetic system, i.e. high HFnu or ANIm, due to the sympathetic depletion in COVID-19 patients are associated with a worse prognosis, higher mortality, and higher IL-6 levels.
